# IoT-based wearable health monitoring device and its validation for potential critical and emergency applications

**DOI:** 10.3389/fpubh.2023.1188304

**Published:** 2023-06-16

**Authors:** Ju-Yu Wu, Yuhling Wang, Congo Tak Shing Ching, Hui-Min David Wang, Lun-De Liao

**Affiliations:** ^1^Institute of Biomedical Engineering and Nanomedicine, National Health Research Institutes, Zhunan, Taiwan; ^2^Program in Tissue Engineering and Regenerative Medicine, National Chung Hsing University, Taichung, Taiwan; ^3^Graduate Institute of Biomedical Engineering, National Chung Hsing University, Taichung, Taiwan; ^4^Department of Electrical Engineering, National Chi Nan University, Puli, Taiwan

**Keywords:** COVID-19, epidemic prevention and isolation, physiological parameter monitoring, Internet of Things, smartwatch

## Abstract

The COVID-19 pandemic brought the world to a standstill, posing unprecedented challenges for healthcare systems worldwide. The overwhelming number of patients infected with the virus placed an enormous burden on healthcare providers, who struggled to cope with the sheer volume of cases. Furthermore, the lack of effective treatments or vaccines means that quarantining has become a necessary measure to slow the spread of the virus. However, quarantining places a significant burden on healthcare providers, who often lack the resources to monitor patients with mild symptoms or asymptomatic patients. In this study, we propose an Internet of Things (IoT)-based wearable health monitoring system that can remotely monitor the exact locations and physiological parameters of quarantined individuals in real time. The system utilizes a combination of highly miniaturized optoelectronic and electronic technologies, an anti-epidemic watch, a mini-computer, and a monitor terminal to provide real-time updates on physiological parameters. Body temperature, peripheral oxygen saturation (SpO_2_), and heart rate are recorded as the most important measurements for critical care. If these three physiological parameters are aberrant, then it could represent a life-endangering situation and/or a short period over which irreversible damage may occur. Therefore, these parameters are automatically uploaded to a cloud database for remote monitoring by healthcare providers. The monitor terminal can display real-time health data for multiple patients and provide early warning functions for medical staff. The system significantly reduces the burden on healthcare providers, as it eliminates the need for manual monitoring of patients in quarantine. Moreover, it can help healthcare providers manage the COVID-19 pandemic more effectively by identifying patients who require medical attention in real time. We have validated the system and demonstrated that it is well suited to practical application, making it a promising solution for managing future pandemics. In summary, our IoT-based wearable health monitoring system has the potential to revolutionize healthcare by providing a cost-effective, remote monitoring solution for patients in quarantine. By allowing healthcare providers to monitor patients remotely in real time, the burden on medical resources is reduced, and more efficient use of limited resources is achieved. Furthermore, the system can be easily scaled to manage future pandemics, making it an ideal solution for managing the health challenges of the future.

## 1. Introduction

COVID-19 spread rapidly to all parts of the world and was out of control due to its strong ability to spread and widespread global trade and travel (1). Almost no country was spared from this disaster. Especially in Italy, the number of patients increased at a breakneck speed, and there was no drug or therapy against the virus when the pandemic first occurred (2). The exceptionally high rate of severe illness and death resulted in a completely overwhelming and almost complete collapse in medical treatment. The number of fatalities increased rapidly, and the world fell into extreme panic.

Since the virus mainly infects the lungs, the primary transmission method is through the respiratory tract. As a result, the transmission tracks include air, droplets, and body contact. Therefore, the transmission tracks must be prevented from being polluted by the virus. Until effective vaccines and treatments are developed, the easiest and quickest solution is to block the virus transmission chain. For people who have not been infected, wearing appropriate protective tools, such as masks, is the essential equipment and requirement to stop the virus from spreading (3, 4). The best way to treat and control infection for infected people is to isolate and observe (5–7). Specialized hospitals with professional medical equipment are necessary for severe patients. The physiological states should be stabilized through supportive therapy and strictly monitored, with the hope that the immune system of a patient can eventually overcome the virus and gradually recover. However, due to the number of patients skyrocketing in the short term, hospitals did not have enough equipment and human resources to fulfill the difficult situation. Therefore, the government could only arrange locations and simple food supplements as basic isolation needs for patients with mild or asymptomatic symptoms. Because of privacy issues, camera equipment cannot be used, and patient situations can be evaluated through only intermittent voice or short face-to-face checkups and confirmation. Currently, using a smartphone as a monitoring tool is a practical method. However, accuracy and effectiveness are challenged by the use of smartphones, particularly with respect to ensuring that quarantined people stay within their allowed places. The advantages and limitations of this method will be further explained in Chapter 2.2. These passive and indirect methods cannot immediately acquire and monitor physical or health status. Quarantined persons will not only suffer emotionally during the quarantine period, but their physical and mental health will be exposed to extremely high risks (8).

Due to the practical problems above, the primary purpose of this research is to develop a system consisting of a wearable device as well as a backend monitoring and warning application that can monitor, upload and warn of the exact locations and essential physiological parameters of mildly symptomatic and asymptomatic patients who are quarantined. To be efficiently carried, miniaturized optoelectronics were used. IoT technology and a mature network environment are embedded for wireless usage, and a backend server and user interface are used to demonstrate the physical status of people who are quarantined (9, 10). The body temperature, oxygenation, and heart rate are monitored because the virus mainly infects the lungs, which can cause lung inflammation and fever. Malfunctions of the lung can also result in lower oxygen levels and a heart rate increase (11). Furthermore, abnormal observations of body temperature, peripheral oxygen saturation (SpO_2_), and heart rate necessitate immediate medical attention. Otherwise, a life-endangering event or irreversible damage may occur.

**Figure 1:**
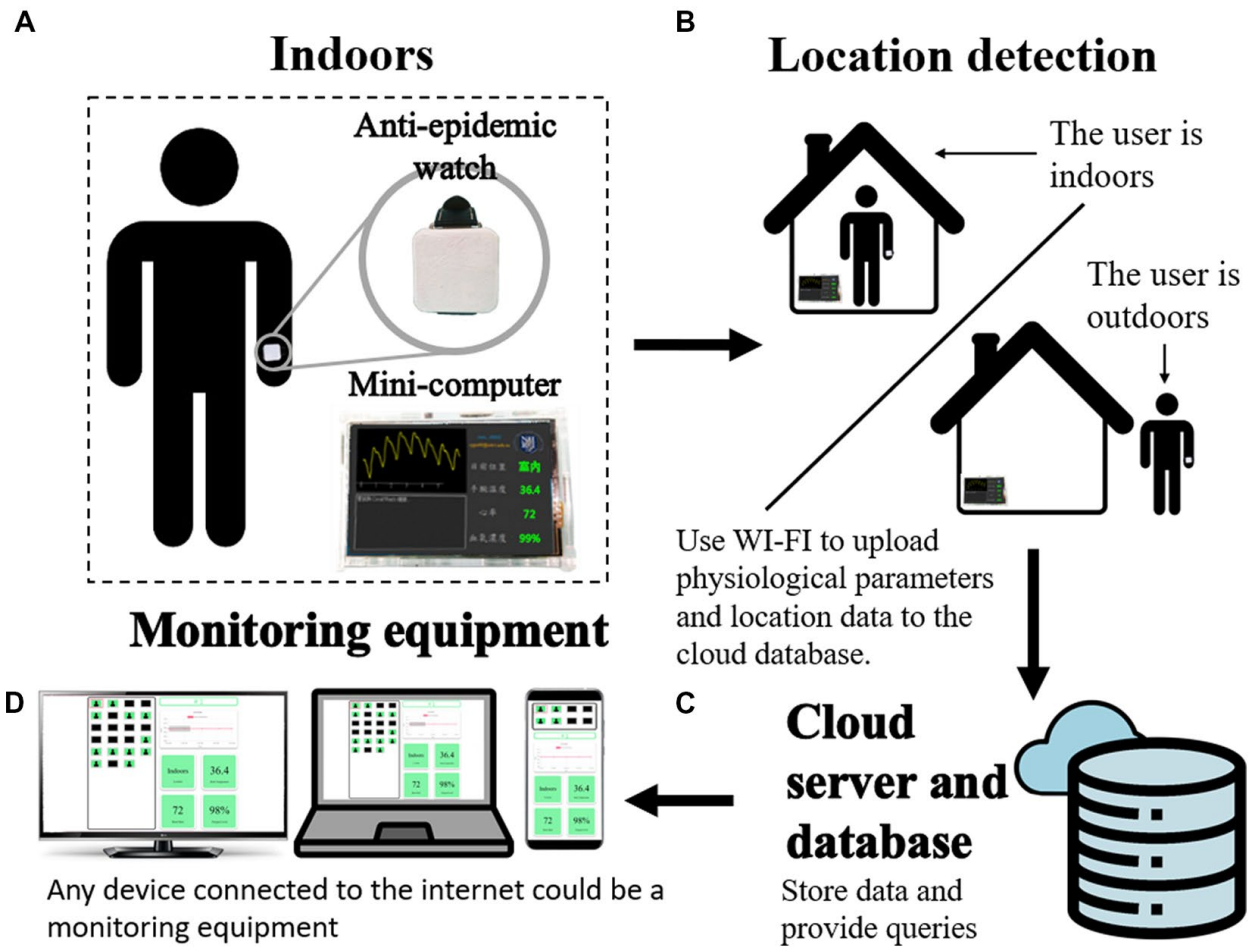
The one-to-many IoT-based wearable health monitoring device system. **(A)** The wearable anti-epidemic watch is on the user, and a mini-computer is fixed in the quarantined place for data collection, receiving, and uploading. **(B)** The combination of an anti-epidemic watch and a mini-computer can detect the location of the user. **(C)** A cloud server and database can receive and store user data. **(D)** The terminal webpage can be displayed on any device.

The system includes a wearable prototype anti-epidemic watch and a set of monitoring systems. Figure 1 shows the overall structure diagram of the system developed in this research. The system consists of three parts. The first part is the anti-epidemic watch, a wearable device worn on the hands of quarantined people, and a mini-computer fixedly installed in a quarantined location, which can receive and show the user status and data, as shown in Figure 1A. Figure 1B shows the anti-epidemic watch combined with the mini-computer as the essential equipment for location detection and the important present physiological state of the person. The second part of the system includes a cloud server for transferring and processing related data and a database system for storing and obtaining all associated data (Figure 1C). The final part of this system, shown in Figure 1D, is the terminal equipment for monitoring the status of isolated individuals. Medical staff can browse an individual’s health status on any device connected to the internet, such as a computer, laptop, or smartphone. If the benefits and expectations of this system can be met, the shortcomings mentioned above can be addressed. Not only can the system provide more effective health management for quarantined individuals in real time, but automatic data uploading to the database and early warning functions will also reduce pressure and burden on health managers and medical staff. A system coupled with statistical data on physiological information can provide an accurate and conducive data set for decision-makers to dynamically adjust the direction of pandemic prevention policy.

This research emphasizes rapid prototyping, thus integrating ready-to-use components and technologies, such as Arduino series microcontroller modules and peripheral sensor modules, PPG sensors, thermistor temperature sensors, a Raspberry Pi single-board mini-computer, local area network connection equipment, free cloud server and cloud database. Self-written software and firmware programs are used together in the prototypes and application designs to meet actual needs as much as possible, as well as a wearable watch with a suitable size, rechargeable and sufficient continuous usage time, and automatic measurement of the critical physiological parameters, location details, and physiological status warnings, and most importantly, economic materials and production costs. Details of the implementation method are discussed in the following chapters. Under these conditions, a prototype of the anti-epidemic watch and IoT environment settings we designed, which we compared to existing simple anti-epidemic devices in the market and other research laboratories, is already very close to practical application, not just in a laboratory research stage, except for a few parts that still need to be strengthened and improved. These are the reasons that this research direction is feasible. It will be possible to convert this system into a commercial transfer model in the future with further optimization in terms of performance, size, and cost.

## 2. Materials and methods

### 2.1. System structure

**Figure 2:**
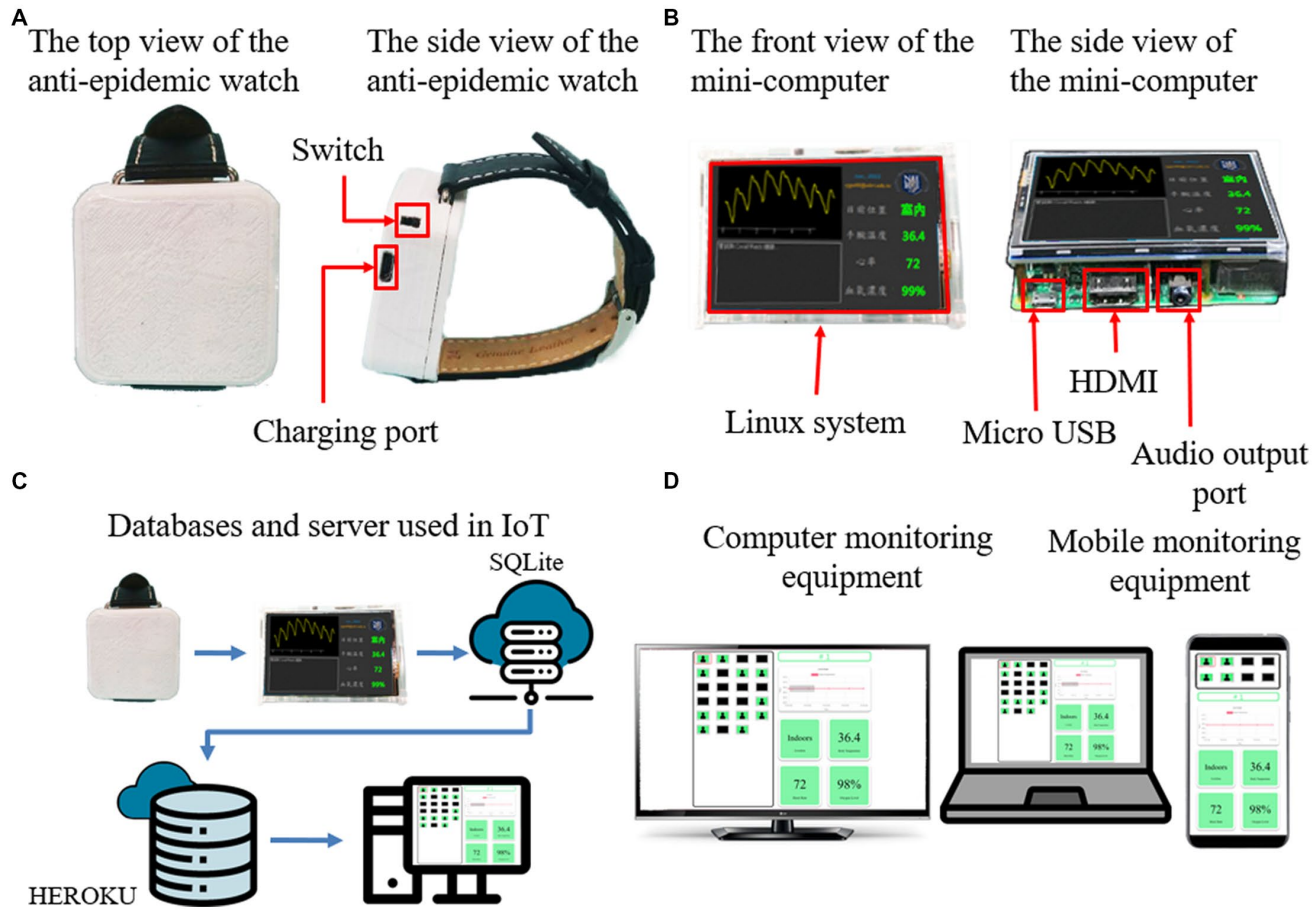
The comprehensive structure of the one-to-many wearable health monitor system. **(A)** An anti-epidemic watch for physiological parameter measurement. **(B)** A mini-computer as a relay station. **(C)** An IoT cloud server and database. **(D)** The terminal webpage can be displayed on different devices.

Figure 2 shows the comprehensive structure of the one-to-many wearable health monitor system, which is composed of an anti-epidemic watch, a desktop mini-computer, and terminal equipment. Figure 2A shows the wearable anti-epidemic watch, with wireless location monitoring and physiological parameter measurement functions. Figure 2B shows the mini-computer with a small screen that can receive wireless signals from the anti-epidemic watch and connect to the internet to upload data to a cloud server and database, shown in Figure 2C, dedicated to the cloud IoT and shows the user’s physical status visualization. Smartphones, tablets, laptops, computers, and large-screen devices in a central control room can be terminal equipment when connected to the internet (Figure 2D). The following factors have been considered from the beginning of the system design. Without redesign or significant modification and with only slight adjustments to the software configuration parameters, the scale of devices and equipment can be flexibly varied according to actual needs. If there are only a few people in quarantine, the correspondingly required quantity of anti-epidemic watches can be operated. In contrast, if more people need to be quarantined, the system can increase the number of anti-epidemic watches in operation and expand the breadth of their deployment. The following chapters introduce how each part is implemented.

### 2.2. Quarantine status monitoring

There must be a particular positioning method to confirm whether an individual has left the quarantine place when under quarantine. Wireless monitoring is inevitable in an isolation room. The most common method in Taiwan is the global system for mobile communications (GSM) signal of a smartphone through multiple base stations to determine the location of an individual (12), which offers advantages and limitations. The advantage is that the GSM signal uses off-the-shelf technology, only requiring a specific software program design, not additional hardware equipment. However, the positioning accuracy is very low due to the limited innate conditions. It can only mark a large-scale rough area, and it is impossible to distinguish whether the current location is indoors or outdoors. A more feasible approach for accurate position control is to use short-distance transmission data communication technology, such as Wi-Fi (13) or Bluetooth (14, 15). These technologies can identify minor distance differences of approximately a half meter (16). However, additional dedicated devices are needed, such as a wearable device to transmit the signals and a host device to receive Wi-Fi or Bluetooth signals and upload the data to the network cloud.

The design concept includes location monitoring and physiological health status measurement functions. More than a pure software application is required to achieve this purpose. The development of exclusive anti-epidemic watches is an inevitable option. The power consumption in anti-epidemic watches is the main influencing factor. Therefore, low-power Bluetooth technology is chosen as the positioning medium. For rapid demonstration, an Arduino Nano 33 BLE (Bluetooth Low Energy, BLE) was used as the main circuit board of the watch because it has built-in low-power Bluetooth circuits and functions, so no additional Bluetooth modules are needed. Arduino’s standard Arduino BLE library was used in writing the firmware program. The BLE module sends an advertising packet every 200 ms. The received signal strength indication (RSSI) of the advertising packet corresponds to the distance from the Bluetooth signal transmitter to the receiver (17). The RSSI can be used to detect the location of a quarantined user indoors or outdoors because the RSSI strength will be reduced significantly by wall attenuation. Moreover, the content of the broadcast packet also includes the BLE Media Access Control (MAC) address, which is a unique value for each BLE module and can be used as a unique identifier for each watch. Therefore, it can detect the specific location of a particular user if multiple devices exist nearby.

### 2.3. Physiological parameter measurement

The aim of this study is to monitor the status of anti-epidemic watch users, and continuously monitoring the physiological index of quarantined users is essential. When health parameters such as body temperature, heart rate, and saturation of peripheral oxygen (SpO_2_) (18, 19) change, the medical staff can observe the status closely and determine the treatment in time. This system, which can simultaneously monitor more than one location state and the physiological state of the users, provides extra protection for the quarantined users. Moreover, the system ensures that the anti-epidemic watch is worn on the wrist of the quarantined person. Therefore, when the patient removes the anti-epidemic watch, data stop being transmitted to the system, and healthcare providers can easily follow up on the reason for removal to fulfill the patient’s needs. Hence, the physiological data cannot be measured once the watch is taken off, achieving a double monitoring function.

A thermal sensor on the watch is embedded to measure the wrist’s warmth. A negative temperature coefficient chip thermistor MF5B with a B value of 3,950 is standard calibrated. The thermistor value measured by the chip is then transmitted to the mini-computer via the Bluetooth signal of the watch (20). The mini-computer host converts the temperature into the corresponding Celsius temperature with a Python program and displays it on the liquid crystal display (LCD) screen. Although the thermistor response speed is relatively slow, it is not a major problem. The wrist is an extremity of the body and is usually colder than the usual locations for this measurement. However, the watch is worn continuously on the wrist, and the continuous body temperature change trend is measured, allowing the health manager to observe the user’s health condition. The trend of body temperature change can sufficiently represent the physiological characteristics of interest. Moreover, it can help to reasonably judge whether the watch is actually worn on the hand, thereby avoiding the disadvantage of using a smartphone as a location monitor. To prevent the user from leaving his or her phone in a room and exiting the allowed region, multiple geolocation confirmations are essential. Therefore, in this system, the changes in body temperature are emphasized rather than the actual body temperature values.

A photoplethysmography (PPG) sensor on the back of the anti-epidemic watch, with red light and infrared light waveforms, can detect the heart rate and blood oxygen concentration (SpO_2_) of the user (21). The data are calculated using a C++ program in the Arduino Nano 33 BLE on the watch and transmitted to the mini-computer via the Bluetooth signal of the watch. The mini-computer can display the heart rate and blood oxygen concentration values on the LCD screen in a visualization line graph. The PPG sensor module in the anti-epidemic watch is a MAX30102 heart rate and blood oxygen concentration sensor produced by Maxim Integrated. There is a program in the Arduino library that provides corresponding support for calculating the heart rate and blood oxygen concentration. Although the correct blood oxygen concentration value still needs to be calibrated, the presentation of the blood oxygen concentration in this plan is mainly to verify the feasibility of the study.

### 2.4. IoT environment

An IoT device should have adequate properties. First, it should be able to pair with the anti-epidemic watch. Second, the data should be received from the watch and uploaded wirelessly to the cloud server and database. Finally, proper display equipment is required to demonstrate the data receiving and transmitting status. Therefore, the mini-computer in the system is an appropriate device. It can be paired with the watch for BLE wireless signal transmission. Furthermore, it has a small LCD screen that can display the Bluetooth connection status and the current physiological parameters. Moreover, it can connect via wireless Wi-Fi or wired LAN to upload the user health index to the cloud database. The mini-computer is a relay platform between the anti-epidemic watch and the cloud server and database, which focuses on a compact size, low price, high reliability, low power consumption, and suitability for long-term operation. Thus, a Raspberry Pi 3B with a 16 GB microSD memory card is selected as the main central processing unit and the computer memory. A Waveshare 3.5-inch RPi LCD is employed as the LCD screen. The combination of these components fully meets the requirements. The software program is written in Python to shorten the development timeframe, and the GUI display interface is developed with the PyQt library. The operating system is Raspberry Pi v10 Buster OS because the v11 Bullseye OS has compatibility issues with the Bluetooth library. The mini-computer can be operated by connecting and turning on the power. It can execute relevant monitoring programs coupled with an anti-epidemic watch in advance and establish a connection spontaneously. There is no need for a manual keyboard or mouse operation. When power is restored after a failure, it can resume work automatically. The mini-computer is a successful IoT device due to its easy control as an ordinary electrical unit.

The cloud server and database need to meet various requirements. When the whole system is operated, the server is not only for a simulation and display within the local network and host. Thus, a powerful server is necessary. However, this paper is still in the development and assessment stage, and free resources available on the internet are utilized. The FastAPI programming framework is used as the development tool for cloud servers because FastAPI is embedded in Python and can operate in a Python virtual environment. Moreover, Python is a programming language with high scalability, convenience, and rapid development. FastAPI must be executed as a platform as a service (PaaS). We explored some free cloud resources, but they failed to meet the specified requirements. Therefore, a self-built server is employed as the IoT environment server. In addition, a web browser user interface (UI) client is built using standard HTML and CSS, and a self-written JavaScript program is added to meet the data requests and respond to the server. The existing UI design is relatively simple, and the purpose is to examine the feasibility and functionality. When this system is officially operational, the virtual environment will be built properly. The browser UI will beautify and strengthen its integrity, and a dedicated server for privacy and stability considerations will be employed.

## 3. Results and discussion

### 3.1. Completed IoT-based wearable health monitoring device and system

A prototype of the one-to-many IoT-based wearable health monitoring device system has been completed according to the design concept introduced above. An anti-epidemic watch, a mini-computer, and a web system have been established. The system can be connected to the network to allow the location and physiological status to be collected from the anti-epidemic watch, which is only for measurement and has no display or storage function. Then, the mini-computer can collect data from the anti-epidemic watch, calculate the raw data to obtain the actual physiological parameters, and show them as line graphs on the LCD. The mini-computer can also upload the preprocessed data to the cloud database. The cloud database can properly store relative data. Finally, the terminal website can request data from various anti-epidemic watch users from the cloud server and display the location and health status to the administrator, who can use the data for health management or further research use.

**Figure 3:**
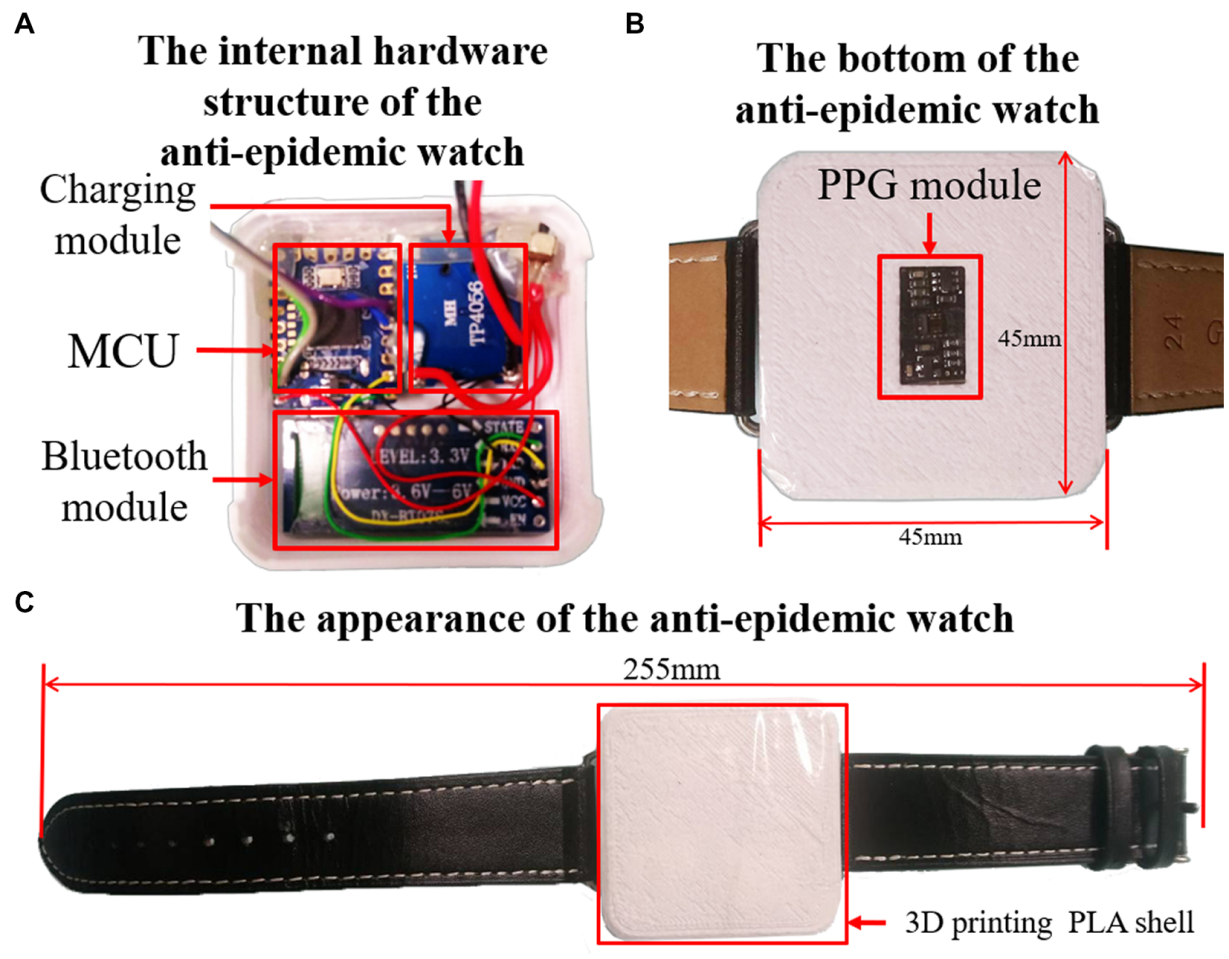
The detailed structure of the anti-epidemic watch. **(A)** The internal hardware structure of the anti-epidemic watch. **(B)** The bottom view of the anti-epidemic watch. **(C)** The appearance of the anti-epidemic watch.

The appearance of the anti-epidemic watch prototype in Figure 3 is straightforward and elegant. It has hidden three-color LED lights on the front to display the current operating status, representing the charging status, connected or disconnected, and standby or measuring. There is a Micro USB charging port and a power switch on the side of the anti-epidemic watch. Figure 3A shows the internal hardware structure of the anti-epidemic watch. It consists of a micro control unit (MCU) module, Bluetooth module, power switch, charging module, and rechargeable 750 mAh lithium-ion battery. At the bottom of the device is a PPG and temperature sensor module to measure body temperature, heart rate, and blood oxygen concentration (Figure 3B). The shell of the anti-epidemic watch is self-designed and constructed using a 3D printer with white polylactic acid (PLA). As shown in Figure 3C, the shell is a 255 mm square, similar to a sports watch, and can be comfortably worn on the wrist.

**Figure 4:**
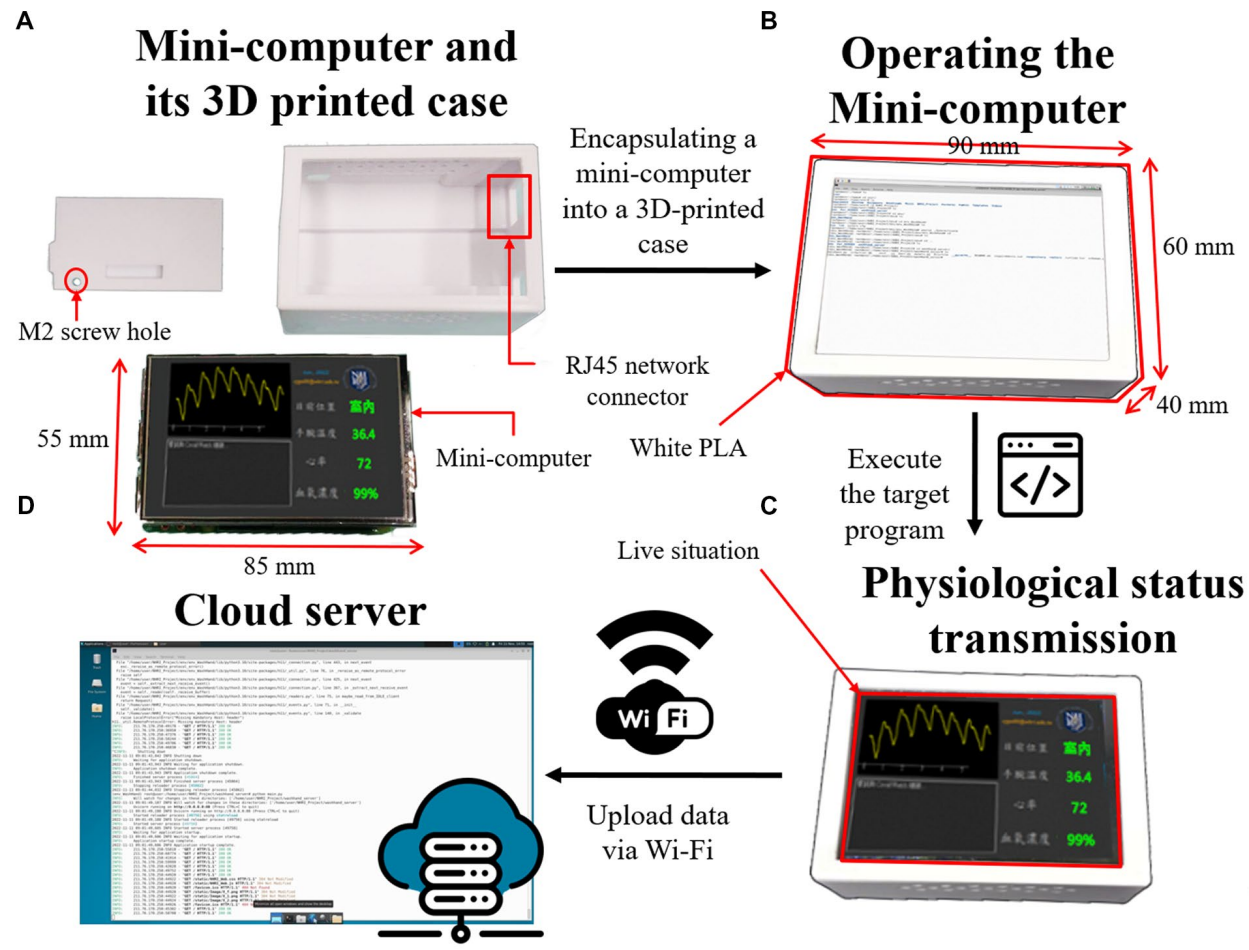
The detailed structure of the mini-computer. **(A)** The mainframe case and the single motherboard of the mini-computer with an LCD. **(B)** The operating mini-computer is encapsulated in the case. **(C)** The LCD of the mini-computer displays the current health and location status of the watch user in real time. **(D)** The data can be uploaded to a cloud database through Wi-Fi.

The structure and functions of the mini-computer are shown in Figure 4. The size of the whole mini-computer is only 90 mm x 60 mm x 40 mm, which is slightly thicker than the Raspberry Pi 3B single motherboard, which is 85 mm x 55 mm x 30 mm. The mainframe case of the mini-computer is the same as the anti-epidemic watch, which is tailor-made using a PLA 3D printer, with an RJ45 network connector hole for wired LAN and an M2 screw hole to fix the motherboard and the LCD display (Figure 4A). The case encloses the mini-computer, as shown in Figure 4B, which can be installed in the quarantine room for data transmission. The operating system of the mini-computer is Linux for Raspberry Pi–the Buster OS, the software is written in Python, and the GUI is developed with the PyQt library. The operating system is sufficiently stable and does not easily crash. A microSD replaces the hard disk because it consumes less power and is much tougher. It can pair with the anti-epidemic watch automatically through Bluetooth and receive data from it. The LCD of the mini-computer can display the current health and location status of the watch user and show the body health index in a visualized pattern in real time (Figure 4C). Furthermore, the data are automatically uploaded and stored in the database of the cloud server through Wi-Fi or LAN at regular intervals, as shown in Figure 4D. Therefore, the mini-computer is very reliable for continuous operation. Moreover, the watch user can observe the physiology index from the mini-computer for psychological comfort.

**Figure 5:**
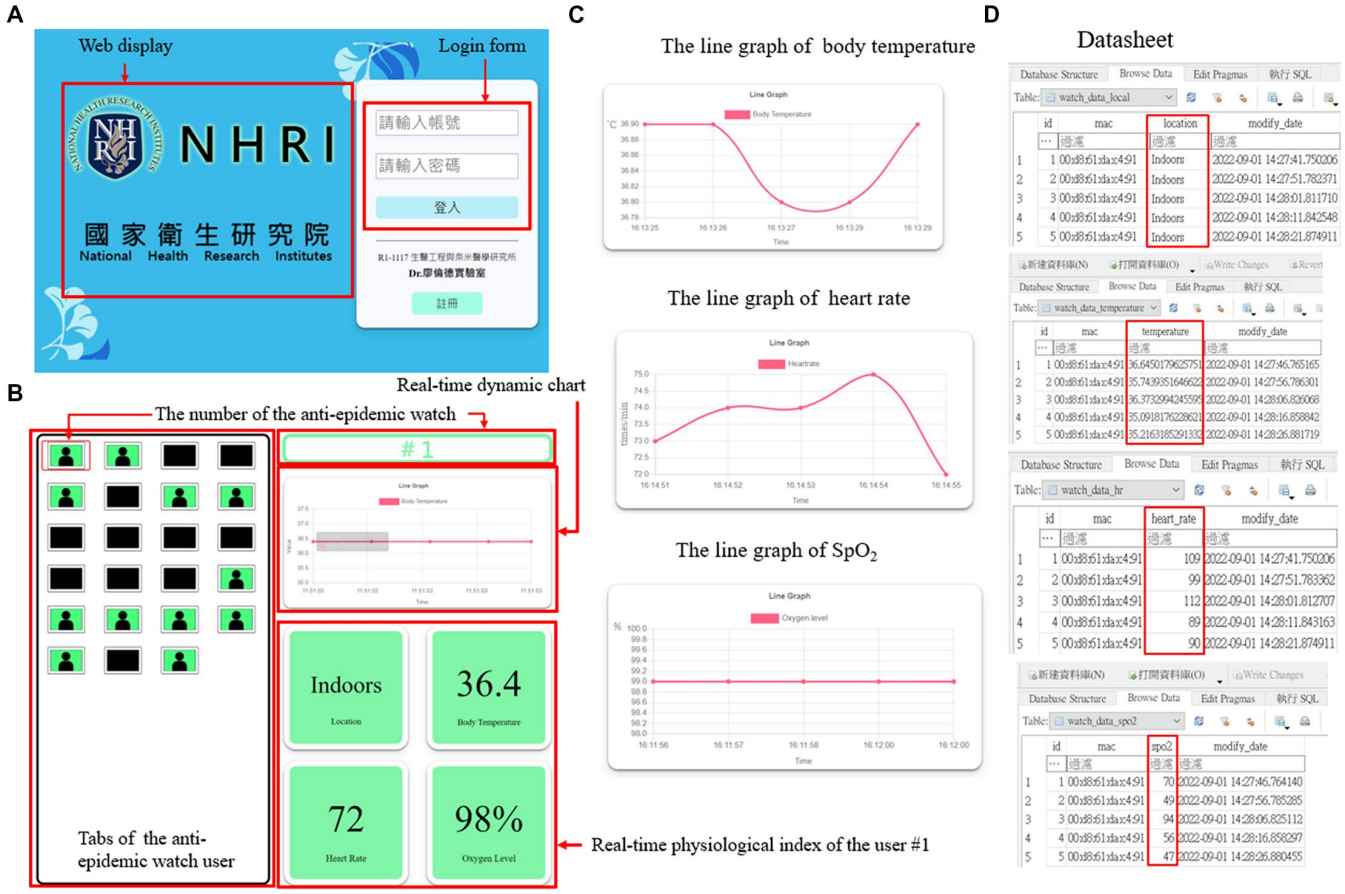
The structure of the terminal webpage on a desktop computer or a laptop. **(A)** The home page of the terminal webpage with login function. **(B)** The user interface (UI) of the terminal webpage. **(C)** The real-time line graph of various physiological indices of a specific user. **(D)** The datasheet of the location and different physiological indices of a particular user.

**Figure 6:**
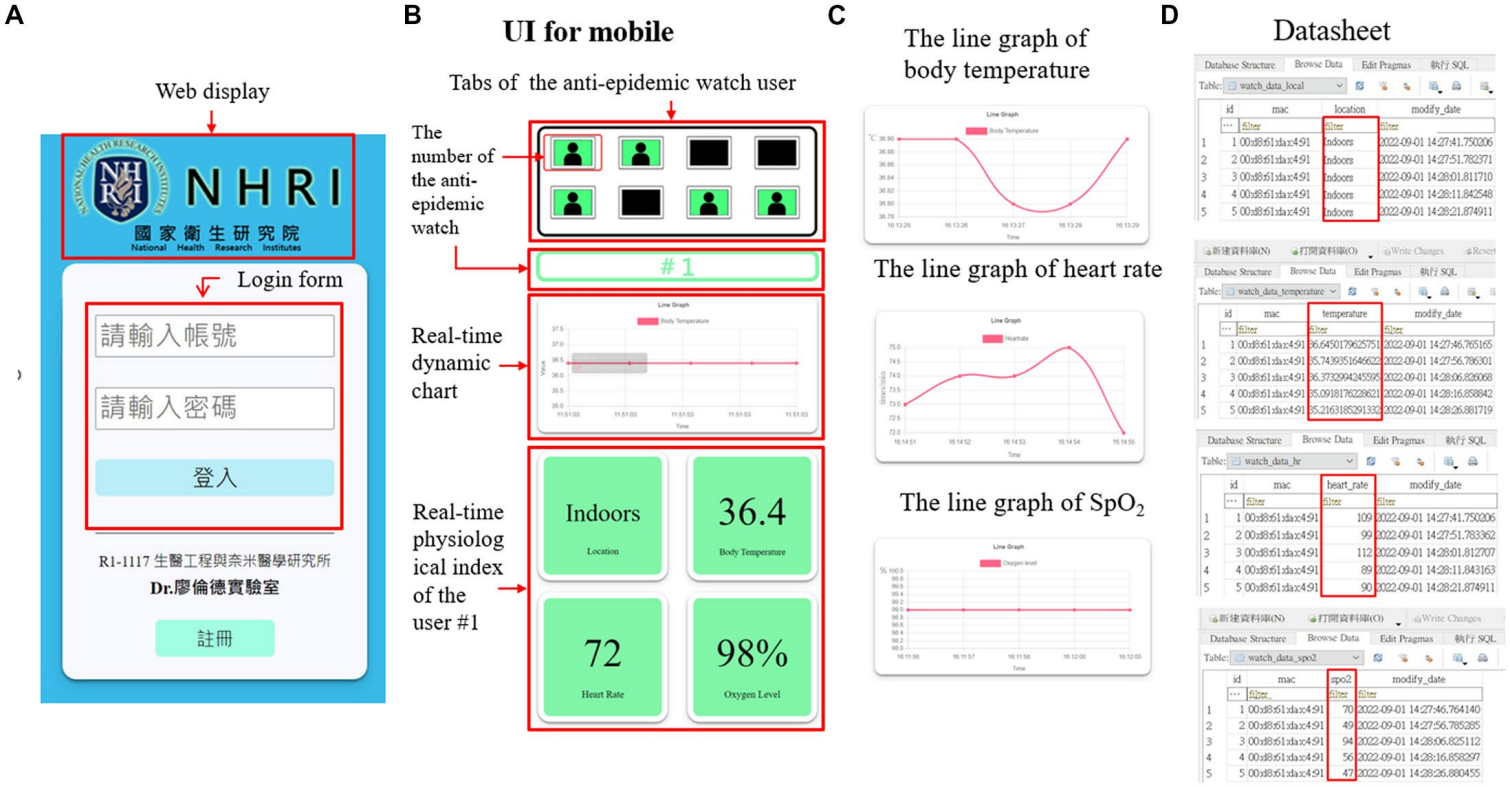
The structure of the terminal webpage on a mobile device. **(A)** The home page of the terminal webpage with login function. **(B)** The user interface (UI) of the terminal webpage. **(C)** The real-time line graph of various physiological indices of a specific user. **(D)** The datasheet of the location and different physiological indices of a particular user.

The webpages on the terminal can be displayed on a desktop computer, a laptop (Figure 5), a mobile phone (Figure 6), and any device with an internet browser. Responsive web design (RWD) technology has been embedded in the webpages, making the webpages fit into any browser and automatically typeset. The RWD can display web pages with a dynamic adjustment to the various components according to the different screen sizes and provide the best user experience. The essential user functions, such as registration and login, have been completed. Therefore, a user can have a better experience with this UI. The login page shown in Figures 5A, 6A of the system can only be accessed through authorized health managers with passwords, which effectively protects personal data privacy.

The webpage UI has three main functions, tabs for the anti-epidemic watch user, a real-time dynamic chart, and a real-time user physiological index, which are shown in Figures 5B, 6B. The tabs of the anti-epidemic watch user can display the situation of every anti-epidemic watch user. The real-time physiological index shows the locations, body temperature, heart rate, and SpO_2_ level of a specific anti-epidemic watch user. The real-time dynamic chart default is the body temperature. However, after clicking the index button, the dynamic chart will change to fit the relative index. Except for the location information, other indices can be visualized as a line graph (Figures 5C, 6C). All user information is stored in a cloud database as a datasheet, as demonstrated in Figures 5D, 6D, and can be checked and analyzed when necessary.

**Figure 7:**
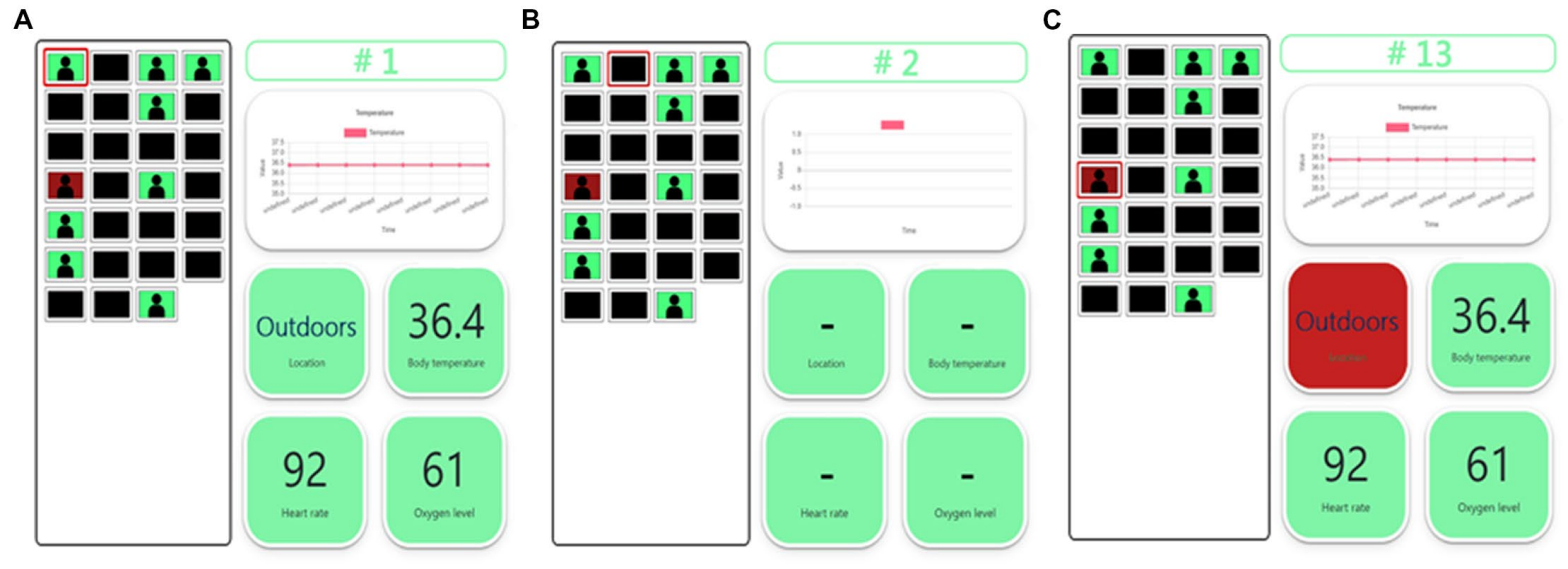
The UI of the terminal webpage. **(A)** The user of the anti-epidemic watch is in a normal situation. The background color is all green. **(B)** There is no user for the anti-epidemic watch. The background color is black, and no data are shown. **(C)** The user of the anti-epidemic watch is in an abnormal situation, such as outdoors. The background color in the abnormal status turns red.

A health manager can monitor the anti-epidemic watch user tabs, as shown in Figure 7. Every anti-epidemic watch has a number, which matches a tab on the terminal webpage. When a user watch is paired with the mini-computer in the room, the data are detected and transmitted. Suppose the user is indoors and in a healthy condition. In that case, the window background color of the terminal webpage in the tab is green, and every background color of the health index button is green, as illustrated in Figure 7A. If there is no watch paired with the mini-computer and no data accessed from the anti-epidemic watch, the window will turn dark, and none of the buttons can be clicked (Figure 7B). However, if the anti-epidemic watch user has some health issues, such as a fever, lower oxygen level, or abnormal heart rate, the green background on the tab will turn red, as shown in Figure 7C. Therefore, authorized health managers can rapidly check the current physiological status related to the personal health of every quarantined person at any time. It is easier for the health manager to quickly note who has an undesired situation. Then, the relative buttons can be clicked, the real-time data will be shown, and the status of the specific user can be confirmed and the abnormal period tracked. For example, if an anti-epidemic watch user has a fever, the background of the user tabs and the body temperature index turn red. Real-time body temperature will be displayed, and the health manager can check how long the user has had a fever and prepare suitable medicine for treatment. This system can help solve problems expeditiously to prevent regrets and decrease the pressures on health managers through an easy-to-understand system webpage UI.

The PPG sensor on the anti-epidemic watch can detect the body temperature, heart rate, and blood oxygen concentration, which is a reliable method for measuring the physical metrics of a watch user. The thermistor MF5B sensor can precisely measure body temperature in this wearable health monitoring device. The temperature range displayed on the mini-computer and the terminal is set from 30.0°C to 42.0°C, and normal body temperature is from 34.7°C to 38.0°C, depending on which part of the body is measured (20, 22). In addition, the PPG sensing element can measure the heart rate and SpO_2_. The data can be transmitted once per second from the anti-epidemic watch to the mini-computer and then to the database, and then the data can be requested from the server and shown on the system webpage. The heart rate range on the anti-epidemic watch sensor is 50 to 200 times per minute, while the standard resting heart rate is only 60 to 85 times per minute and is no more than 100 times per minute when people are at rest (23). The normal SpO_2_ range is approximately 75 to 100%. A patient should be treated immediately when the SpO_2_ is lower than 60% (24).

**Figure 8:**
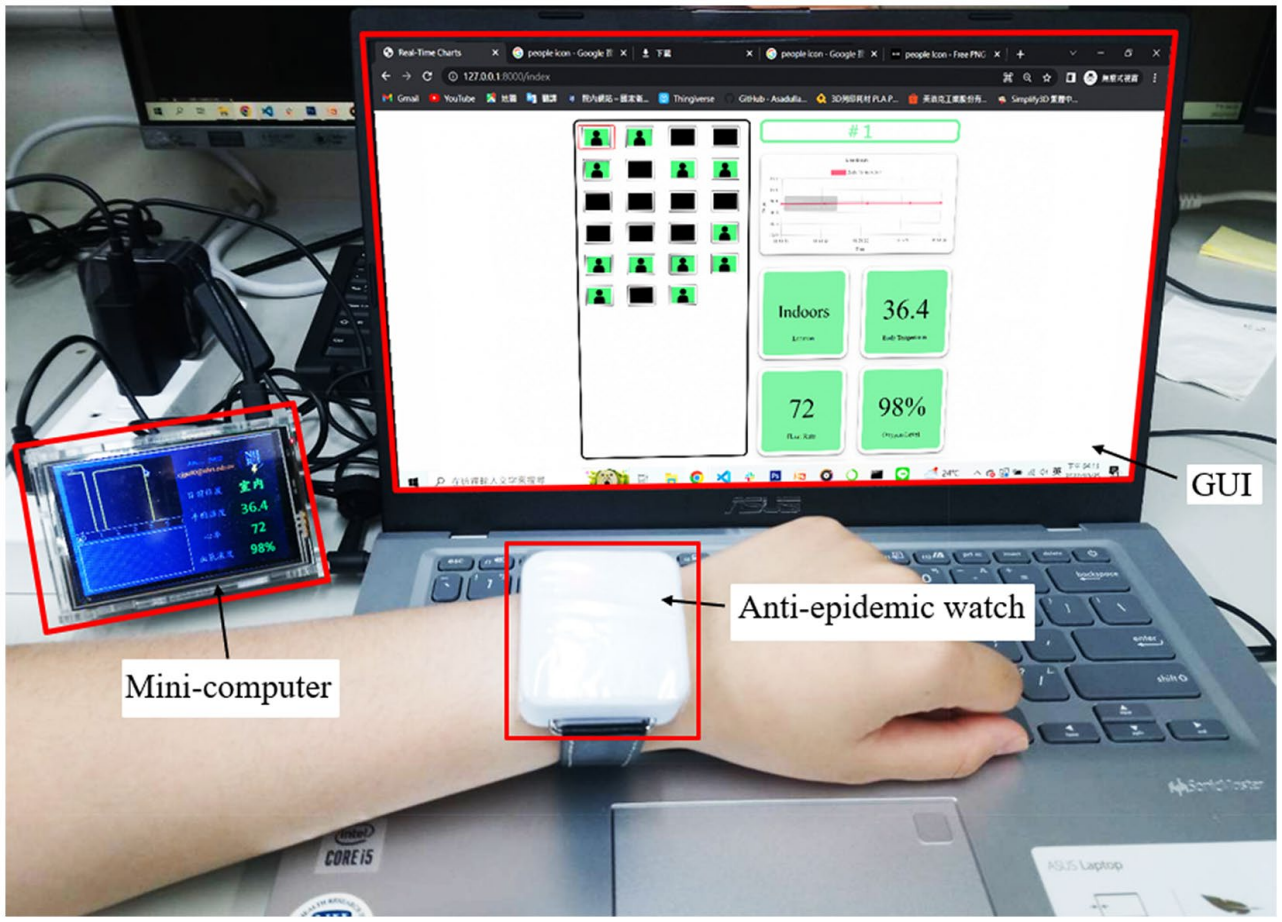
Demonstration of the one-to-many IoT-based wearable health monitoring device system. The data were collected by the anti-epidemic watch, and the mini-computer received the data and then uploaded it to the cloud server and database. The terminal webpage requested the data from a specific user and displayed it in real time. The demonstration is recorded in Supplementary Video S1.

All the physical sensors embedded in the anti-epidemic watch function in a reasonable range. The actual operation of the overall system is shown in Figure 8 and recorded in Supplementary Video S1. The anti-epidemic watch is worn on the wrist of a quarantined user, and the mini-computer is connected to the watch through Bluetooth to transmit instructions and data. The LCD screen of the mini-computer displays the current isolation status and physiological parameters. The mini-computer sends these data sets to a dedicated server through Wi-Fi, which stores them in a database. The monitoring terminal system webpage UI on a browser dynamically displays all relevant information of each current watch user. The heart rate and SpO_2_ of a user are approximately 85 times per minute and 99%, respectively, meaning that the user is in a regular physical status. The background of the windows, which represent all physical indices and the location, is green. The body temperature of the user is approximately 36.5°C to 37.2°C, which is illustrated as a linear graphic on the LCD of the mini-computer, and from the terminal website, the body temperature of the user can be observed in real time.

**Figure 9:**
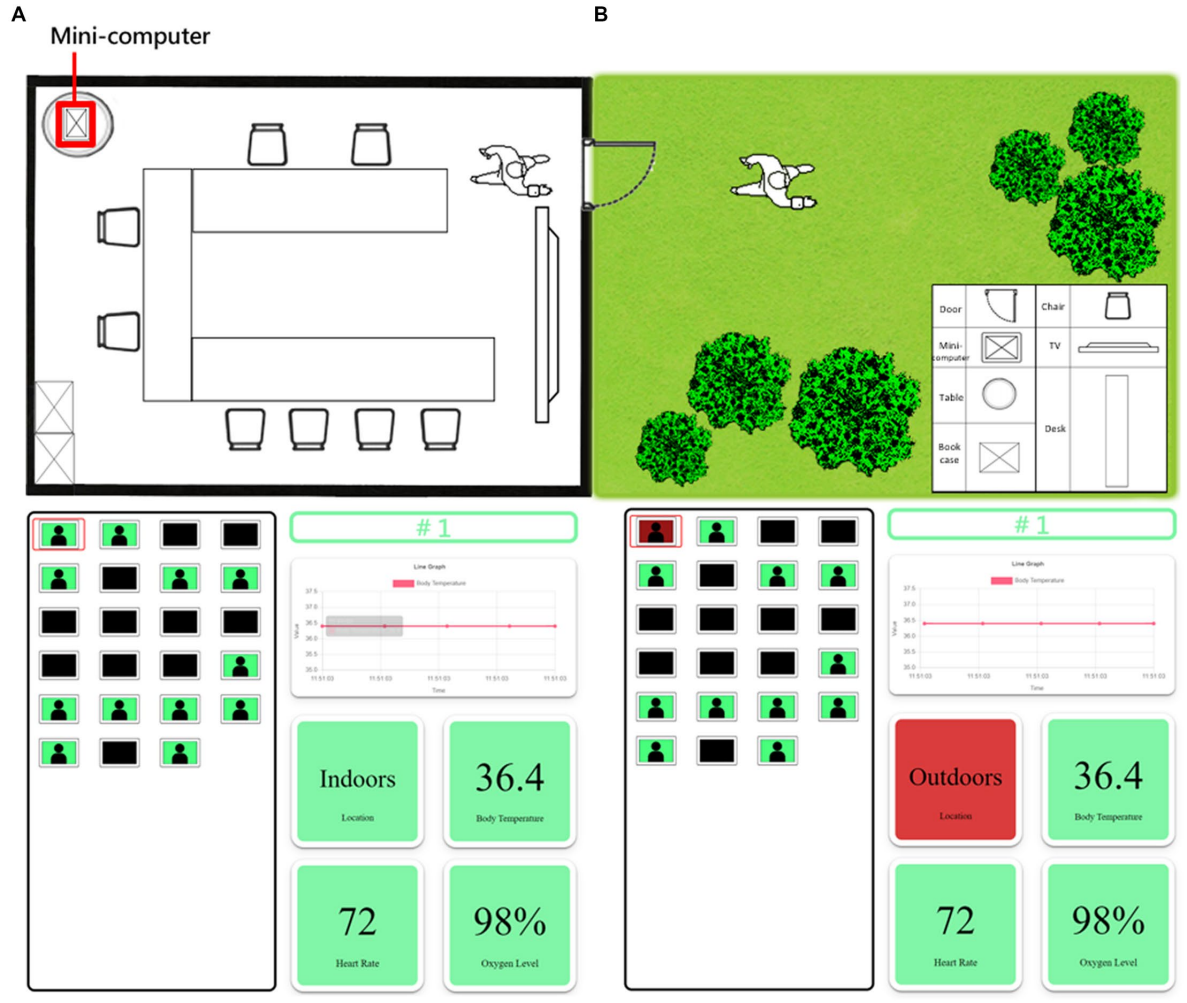
Demonstration of the actual use of the anti-epidemic watch. **(A)** When the user of the anti-epidemic watch is indoors and healthy, the mini-computer and the terminal webpage show everything is in order. The demonstration is recorded in Supplementary Video S2. **(B)** When the user of the anti-epidemic watch is outdoors, the mini-computer and the terminal webpage immediately show the abnormal situation, and the related index is red. The demonstration is recorded in Supplementary Video S3.

System testing of the one-to-many wearable health monitor is demonstrated in Figure 9 and recorded in Supplementary Videos S2, S3. When the anti-epidemic watch user is indoors, as shown in Figure 9A and Supplementary Video S2, the mini-computer in the quarantined room can receive the personal user information from the anti-epidemic watch accurately and show a line graph of the body temperature and the other physiological indices in numbers in real time on its LCD. Watch users can also observe health information themselves from the LCD of the mini-computer. The terminal webpage also shows that all user statuses are healthy by displaying a green background. In contrast, when the watch user goes outside, as shown in Figure 9B and Supplementary Video S2, the situation makes the mini-computer unable to receive a sufficiently strong signal. The location status of the watch user, shown on the mini-computer, changes from indoors to outdoors, and the information is transferred to the terminal. The method of judging whether the current location of the monitored person is indoors or outdoors considers whether Bluetooth can be used to transmit data. When the user is active and indoors, the Bluetooth signal strength is generally above −60 dbm, enabling smooth connection with the mini-computer for the exchange of data and messages. When the user is outdoors, due to the strong attenuation of the Bluetooth signal caused by building walls, its strength will quickly drop below −90 dbm. At this time, connecting and transmitting data is impossible, so it is judged that the user has stepped outside. The tag of the terminal webpage, which is related to the specific number of the watch user, turns red directly. When the tag is clicked, the background of the location index will display outdoors. If something goes wrong, the health manager receives the information that something has happened to the user and can check it quickly and easily. Thus, the medical staff can respond to the situation promptly. Overall, the original goal of this plan has been achieved.

### 3.2. Parts to be improved and future directions

There are a few problems we need to address. One problem is that the watch’s power consumption and continuous use time are insufficient. The Arduino IDE is used to develop the watch firmware, which leads the Arduino Nano 33 BLE microcontroller board not to enter the power-saving mode that the control board initially has under this platform environment. This situation makes the actual power consumption approximately 9.5 mA when only turning on the BLE and temperature sensor with the PPG module is turned off. The operating time of the watch is only approximately 78 h for a 750 mAh battery before recharging. When the PPG sensor is turned on intermittently, the available time may only be approximately 2 days. When the PPG sensor is turned on, it requires approximately 20 mA. The problem can be solved by using the native Arduino Nano 33 BLE development tool to enable the power-saving mode. However, more effort must be put into programming design, and subsequent versions will improve the results.

Another difficulty is that the PPG sensor module, which is for heart rate and blood oxygen concentration measurements, cannot fit the needs of a wristwatch. The existing PPG sensor module is designed for red light and infrared light measurement, which is more precise for SpO_2_ and heart rate measurement. Compared to other commercially used wrist-type PPG sensors, which use green light reflections, the absorptivity of hemoglobin in green light needs to be higher and more accurate (25). However, both PPG sensor signals are minor but still usable for measuring when the sensor is worn on the inside of the wrist. The signal is weaker when the watch is worn in the proper position. The combination of green light and infrared light can be adopted to solve this problem. The reason might be the insufficient brightness of the red and infrared light in the modules, and there is no ready-made high-brightness PPG module for purchase and testing (26, 27). Thus, the present speculation cannot be confirmed. The high-brightness PPG module development process must be improved.

The current communication method” n th’ network between the browser and the terminal server equipment is implemented using a polling server, which is inefficient and wasteful of network transmission resources. Nonetheless, it is acceptable for a few monitoring individuals and verifying feasibility. However, when the number of monitoring patients continually increases and polling is too frequent in a short period, the server will inevitably be exhausted and overwhelmed. Eventually, the server will lag and then crash. The only solution is to adopt a new communication architecture, such as Web Socket, which will significantly reduce the server bandwidth requirement and burden. Thus, a dedicated server and a new communication protocol are needed.

### 3.3. Comparison with results obtained using other methods

This one-to-many wearable health monitoring system is innovative because no existing health monitoring system can monitor the location and multiple physiological parameters of a patient simultaneously and support a monitoring terminal through IoT technology. Several existing systems can track the health statuses of quarantined individuals. One system was designed by Hoang (28), and another was designed by Lim (29). Compared to the device from Hoang, the body temperature and user activities can be detected. Although the IoT technology is embedded, only an easy point-to-point function is applied in the system. There is no dedicated cloud server with a database to provide monitoring functions for multiple users. The wearable device only lasts for 45 min when fully charged. It can lose the capability to monitor the location state if the appliance cannot be worn for a long time. However, the design of the device is regarded as the original level due to the irregular jumper wires in the machine. Therefore, although the IoT part of the system is well designed, the whole system is still an experimental mockup. Therefore, there is a considerable distance from an actual application (30). Furthermore, the wearable watches created by Lim can only detect user location information.

In addition, the system designed by Cruz (31) has the functions of detecting location, body temperature, and blood oxygen concentration in addition to cough detection, making it highly practical. A combination of Global Positioning System (GPS) and LoRa technology is used in this system for data transmission, enabling the transmission of information over long distances. However, GPS can only be used outdoors, and its accuracy can be low indoors. Furthermore, when quarantined, a user will stay in the same room for several days. Thus, the location monitoring function of this system could be less suitable for quarantine; indeed, the intended use of this system differs from the intention in our study, for which indoor location monitoring is needed. Agriesti (32) also designed a device that detects heart rate, blood oxygen concentration, and posture. The location monitoring function, which is critical during a pandemic, is absent. This system is more like a health bracelet. In contrast, the system we designed is fundamentally based on position monitoring and additionally includes the function of monitoring critical physiological parameters. Therefore, there are apparent differences between Agriesti’s system and ours. Furthermore, there are considerable similarities between the systems created by Jie Wan (33) and Agriesti. Both system design concepts focus on health status detection and monitoring. Because these systems follow older designs and are not explicitly intended for isolated location monitoring, the sensors are not integrated into a single wearable but rather are scattered across different body parts, which may be inconvenient for users. Finally, the system designed by Nizar (34) is similar to the system from Cruz. Body temperature, heart rate, blood oxygen concentration, cough, and location are detected by both systems, but user location detection is again based on GPS technology. Therefore, both systems are more applicable for outdoor usage. These systems are less suited to the situation in which individuals are quarantined at home. Compared with the devices and the system designed in this article, which focuses on the centralized isolation and monitoring principles of the public health system, the construction methods for users are enormously different.

In contrast, the system designed in this study considers many necessary factors, and the need is as close as possible to the actual application. Wearables with an IoT environment and a cloud-monitoring terminal, which can control the location and health status of many users, are the most significant difference between other designs and ours (30). Therefore, our project seems more practical than other existing systems.

## 4. Conclusion

In conclusion, the COVID-19 pandemic has highlighted the urgent need for more advanced tools and technologies to manage future outbreaks. This study proposes an Internet of Things (IoT)-based wearable health monitoring system that can remotely monitor the physiological parameters of quarantined individuals in real time. The system can significantly reduce the burden on healthcare providers and help manage the COVID-19 pandemic more effectively. It can also be used as a proactive measure to prevent the spread of future pandemics. A one-to-many system, comprising an anti-epidemic watch and a mini-computer, has been designed to fulfill the insufficiency of existing epidemic prevention tools. It can passively prevent quarantined individuals from going out, block the virus tracks, and avoid the spread of the virus, thereby protecting public health. The system checks the body temperature, heart rate, and blood oxygen concentration at any time, and both a healthcare provider and the anti-epidemic watch user can observe the status. While the world gradually lifts the blockade, quarantine demands have been dramatically reduced. However, this project of a one-to-many monitor system will continue to be developed and improved to overcome known problems and difficulties. For instance, the battery capacity of the anti-epidemic watch, the PPG module, and the IoT design, which are factors of the next-generation design, must be considered. Therefore, requesting a dedicated server or virtual environment is urgent to provide more flexible server programming requirements. Although funds and human resources are limited, work on the second-generation anti-epidemic watch is focused on software and hardware design, and the functions of the cloud part of the IoT are also gradually expanding. A system will be developed to transfer data to multiple types of smartwatches and view information straight forward on the watch in the future. In addition, abnormal state warning functions will be enhanced, such as better monitoring of physiological indices, printing reports, historical data queries, and data analysis. The primary purpose of this ongoing project is to create a system that will eventually become a mature product that can be used for managing not only COVID-19 but also future pandemics effectively.

## Data availability statement

The original contributions presented in the study are included in the article/Supplementary material, further inquiries can be directed to the corresponding author.

## Author contributions

L-DL conceptualized the research. J-YW, YW, CC, H-MW, and L-DL designed the experiments and interpreted the data and wrote the manuscript. J-YW and YW contributed to developing the system and conducted the tests. CC, H-MW, and L-DL supervised the study. All authors contributed to the article and approved the submitted version.

## Funding

This research was supported in part by the National Science and Technology Council of Taiwan under grant numbers 110-2221-E-400-003-MY3, 111-3114-8-400-001, 111-2314-B-075-006, 111-2221-E-035-015, and 111-2218-E-007-019; and by the National Health Research Institutes of Taiwan under grant numbers NHRI-EX108-10829EI, NHRI-EX111-11111EI, and NHRI-EX111-11129EI and by the Ministry of Health and Welfare of Taiwan under grant numbers MOHW 112-0324-01-30-06 and MOHW 113-0324-01-30-11.

## Conflict of interest

The authors declare that the research was conducted in the absence of any commercial or financial relationships that could be construed as a potential conflict of interest.

## Publisher’s note

All claims expressed in this article are solely those of the authors and do not necessarily represent those of their affiliated organizations, or those of the publisher, the editors and the reviewers. Any product that may be evaluated in this article, or claim that may be made by its manufacturer, is not guaranteed or endorsed by the publisher.
